# Two pedigrees with arrhythmogenic right ventricular cardiomyopathy linked with R49H and F531C mutation in *DSG2*

**DOI:** 10.1038/s41439-019-0069-3

**Published:** 2019-08-21

**Authors:** Xuepin Chen, Hui Peng, Chenqing Zheng, Hongmei Zhang, Chao Yan, Huihui Ma, Xiafei Dai, Xiaoping Li

**Affiliations:** 1 Zunyi Medical University, 563000 Zunyi, Guizhou China; 2 Department of Cardiology, Sichuan Academy of Medical Sciences and Sichuan Provincial People’s Hospital, School of Medicine, University of Electronic Science and Technology of China, 610072 Chengdu, China; 3 Department of Cardiology, Tongde Hospital of Zhejiang Province, 310012 Hangzhou, Zhejiang China; 4 Shenzhen Real Omics (Biotech) Co., Ltd, 518081 Shenzhen, China

**Keywords:** Genetic variation, Molecular biology

## Abstract

Arrhythmogenic right ventricular cardiomyopathy (ARVC) presents as the progressive fibrofatty replacement of the cardiomyocytes particularly in the right ventricular wall. Here, we report two cases with ARVC. In family A, the proband carries a *Desmoglein2* (*DSG2*) gene complex heterozygous mutation NM_001943.4:c.146G>A/p.(Arg49His)and NM_001943.3:c.1592T>G/p.(Phe531Cys). In family B, the proband carries a homozygous mutation NM_001943.3:c.1592T>G/p.(Phe531Cys).

Arrhythmogenic right ventricular cardiomyopathy (ARVC:OMIM#610193) is a fatal genetic cardiomyopathy with prevalence rate ~1/2000 to 1/5000^1^. Cardinal manifestations typically involve right ventricular enlargement and dysfunction^2^. The disease is frequently inherited in an autosomal dominant mode with incomplete or complete penetrance, although autosomal recessive transmission has also been reported^3^. The main five cardiac desmosome components (desmoglein-2, DSG2; desmocollin-2, DSC2; desmoplakin, DSP; plakoglobin, JUP; plakophilin-2, PKP2) contribute to 50% or so of symptomatic individuals^4–7^.

Desmogleins are calcium-binding transmembrane glycoprotein components of desmosomes, which are cell–cell junctions between epithelial and myocardial^8^. The *DSG2* *gene* encodes a key cadherin of the cardiac desmosome and the only desmoglein distributed in the cardiomyocytes. *DSG2* is critical for the structural integrity of the intercalated discs, and a lack of *DSG2*-dependent adhesion is a major pathogenic mechanism of ARVC^9,10^. Recent studies have suggested that mutated *DSG2* proteins are incorporated into desmosomes exhibit dominant-negative effects in ARVC^11^. In addition, mutations in *DSG2* display a high degree of penetrance and result in varying levels of disease severity^12,13^. Moreover, patients with multiple desmosomal mutations have shown to have a severe clinical course with more ventricular arrhythmias and a higher frequency of heart failure than subjects with a single mutation^14–16^.

*Family A*: We experimented a male patient, a 7-year-old person (III-2 in Family A; Fig. 1a), presenting with abdominal distension and polypnea. He displayed corroborative evidence of heart failure, such as elevated levels of myocardial damage markers (N-terminal B-type natriuretic peptide [NT-BNP]: 4420 pg/mL, cardiac troponin [cTnI]: 0.046 μg/L). The 24 h dynamic electrocardiogram (DECG) revealed an incomplete right bundle branch block and epsilon waves (Supplemental Fig. S1). The echocardiogram showed enlarged right heart chambers (right atrial, RA = 59 × 57 mm; right ventricle, RV = 36 mm) (Supplemental Fig. S2). Cardiac magnetic resonance (CMR) confirmed RV abnormalities with focal bulges, excessive trabeculations localized in the RV apex. On the basis of these data, ARVC was diagnosed.

**Fig. 1:**
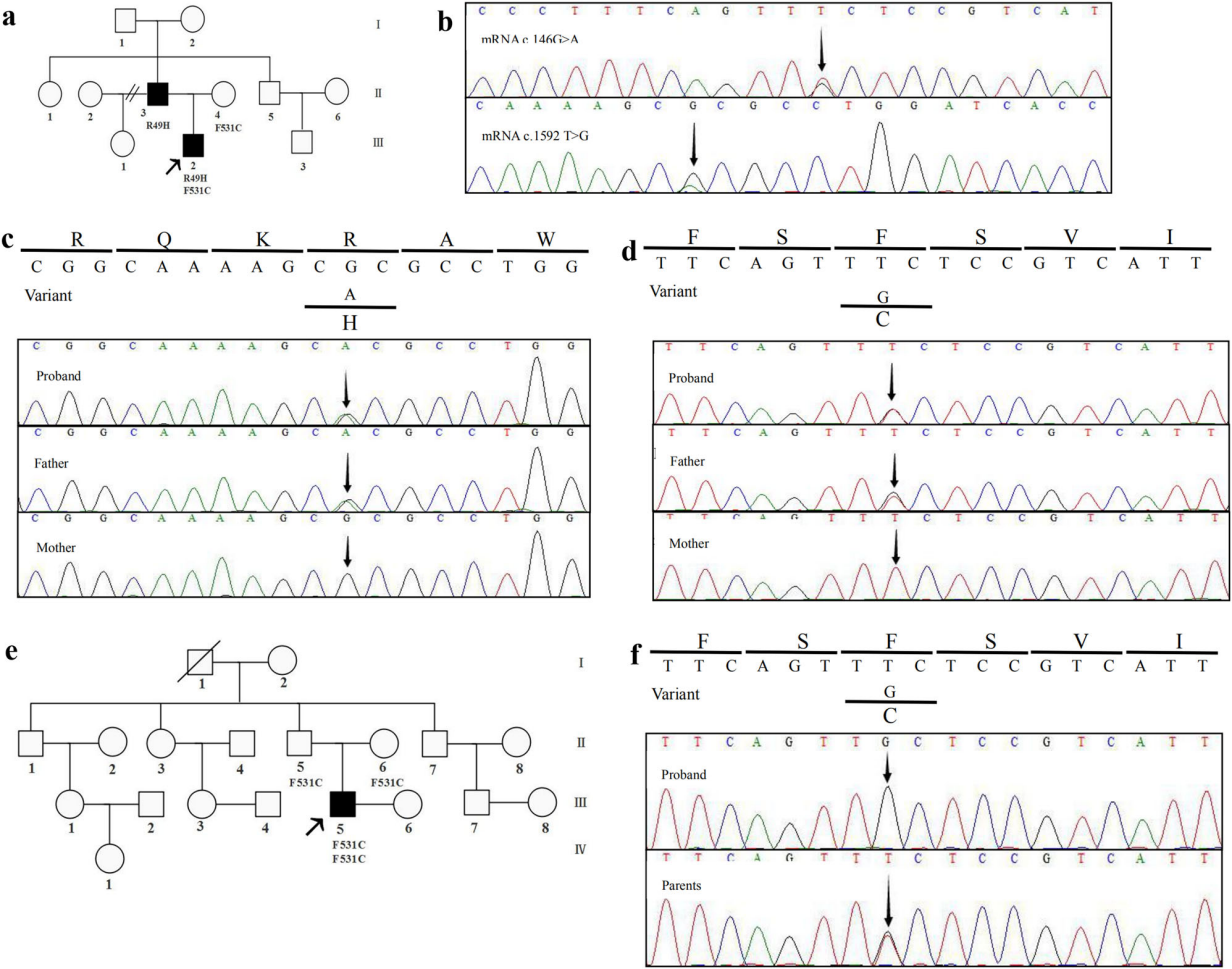
Missense variants in the *DSG2* gene from two Chinese families with ARVC. Pedigrees of the family A (**a**) and family B (**e**). Squares indicate males and circles females, ARVC patients are shown with solid symbols and unaffected with white symbols, carriers with white symbols plus solid point, the arrow indicates the proband. The proband’s (subject III:2) RNA sequence electropherograms are shown in (**b**). The DNA and amino aid sequences of *DSG2* in family A (**c**, **d**) and family B (**f**)

The proband’s father (subject II:3) was first suspected to have ARVC at the age of 43 years when he exhibited an abnormal ECG with inverted T waves from V1 to V3 (Supplemental Fig. S3). His 24 h DCG showed premature ventricular contraction over 500/24 h (1151/24 h). The echocardiogram showed a mildly dilated RV (33 mm) (Supplemental Fig. S3). The proband’s mother (subject II:4) had no clinical symptoms at the age of 44 years and exhibited a normal ECG and echocardiogram.

*Family B*: We also experimented a male patient, a 29 years old (III-5 in Family B; Fig. 1e), who came to medical attention at the age of 23 years on account of recurrent palpitations, chest tightness. The electrocardiogram (ECG) was characterized by inverted T waves in V1–V4 (Supplemental Fig. S5). He exhibited increased right cardiac dimensions (RA = 48*42 mm, RV = 34 mm) with right ventricular aneurysm (11*7 mm) and broadening of the right ventricular outflow tract (RVOT = 38 mm) (Supplemental Fig. S5). CMR confirmed excessive trabeculations in the RV, thinner myocardium in the LV inferior wall and the LV apex, and mild LVEF reduction (49%). Becase of paroxysmal ventricular tachycardia, he was treated by radiofrequency catheter ablation (Supplemental Fig. S4). Based on the findings, the positive diagnosis of ARVC was made. When questioned regarding his family history, the patient mentioned that his grandfather (I-1: Fig. 1e) suffered from sudden cardiac death because of a stroke. His father (II-5: Fig. 1e) and his mother (II-6: Fig. 1e) shows no clinical symptoms and exhibited normal ECG and echocardiograms.

This study was approved by the Sichuan Academy of Medical Sciences and the Ethics Committee of the Sichuan Provincial People’s Hospital Trust. Informed consent were obtained from all individual participants included in this study. Clinical evaluation was based on the revised 2010 Task Force Criteria for ARVC.

Genomic DNA was extracted from peripheral blood samples with the Blood DNA Extraction Kit (Enriching Biotechnology, Shanghai, China). Briefly, using a customized Roche NimbleGen SeqCap EZ MedExome Kit (Roche Diagnostics, Wu Han, China), we targeted and enriched for the exons and the neighboring introns (within 50 bp) of the 130 genes associated with cardiomyopathy. Each quantified library was then loaded on the HiSeqXten platform (Illumina, Germany, Berlin) for next-generation sequencing. The detected variants were annotated and filtered using the following eight databases: ClinVar (https://www.ncbi.nlm.nih.gov/clinvar/), OMMI (https://www.ncbi.nlm.nih.gov/ommi/), RefGene (http://www.ncbi.nlm.nih.gov/RefSeq/), ExAC (http://exac.broadinstitute.org), Ensembl (http://www.ensembl.org), Encode (http://genome.ucsc.edu/ENCODE),1000 Genomes Project (http://www.1000genomes.org, 2014 Oct release), EVS (http://evs.gs.washington.edu/EVS). After filtering the candidates against multiple databases, the retained nonsynonymous single nucleotide variants were submitted to PolyPhen-2, SIFT, and Mutation Taster for functional prediction. Sanger sequencing was used to determine whether any of the remaining variants co-segregated with the disease phenotype in the two families. Sequencing data were compared to the Human Genome Database, and mutation naming followed the nomenclature recommended by the Human Genomic Variation Society (HGVS).

In the proband in family A, p.Arg49His was inherited from his father (Fig. 1b, c). The codon Arg49 is conserved among species (Fig. 2) and the p.Arg49His is predicted to be harmful by PolyPhen-2, SIFT, and MutationTaster. According to the ClinVar (https://www.ncbi.nlm.nih.gov/clinvar/variation/450040/#summary-evidence), this variant is considered as “likely pathogenic” with the frequency of 0.000008 in ExAC. Previously, Awad et al.^8^ reported this variant as a compound heterozygous with the other variant. They predicted that the Arg49His would abolish furin cleavage of pro-desmoglein, thereby disrupting the production of mature, functional protein. Gandjbakhch et al.^17^ also described this variant as a consequence of de novo occurrence. Gaido et al.^18^ suggested that ARVC patients may exhibit extreme phenotypic-variavility in their clinical manifestations, even among patients carrying thep.Arg49His in the same family.

**Fig. 2:**
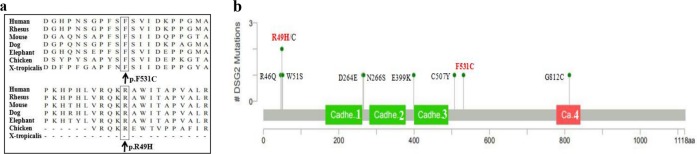
Summary of the *DSG2* variants found in the with ARVC. As determined using Human GRCh37/hg19, NM_001943.4:c.146G>A/p.(Arg49His) and NM_001943.3:c.1592T>G/p.(Phe531Cys) in DSG2 gene were hightly conserved across many species **a**. The DSG2 gene-related pathogenic or likely pathogenic missense mutations reported to date in patients with ARVC, the red ones indicate the mutations identified in the present study. Domains are exhibited with four green rectangles: Cadherin 1: Cadherin domain 165–262, Cadherin 2: Cadherin domain: 281–377, Cadherin 3: Cadherin domain: 400–490, Cadherin 4: Cadherin cytoplasmic region 778–841. The R49H variant at the head all of the domains, while F531C variant between Cadherin 3 and Cadherin 4 domain **b**

We consider that the p.Arg49His is a contributing factor to the severe phenotype of the proband in family A, in contrast with his father who carries the same variant; whereas the p.Phe531Cys inherited from his mother (Fig. 1b, d) would have aggravated the patient’s condition. Lin et al.^19^ reported the homozygous p.Phe531Cys in their cohort. This variant is likely to cause adverse changes in protein structure resulting in protein function changes that may ultimately weaken intercalated discs. Although the frequency of this variant in ExAC is reported as 0.00007, the recent study demonstrated that the p.Phe531Cys is highly prevalent (87%) among Chinese ARVC patients and has full penetrance for homozygous carriers^20^. This indicates that heterozygous carriers of this variant are unaffected.

In family B in this study, we identified p.Phe531Cys as a homozygous pattern (Fig. 1f). Based on the guideline for the interpretation of sequence variants: a joint consensus recommendation of the American College of Medical Genetics and Genomics and the Association for Molecular Pathology, p.(Arg49His) and p.(Phe531Cys) were classified as likely pathogenic, respectively.

In conclusion, we identified homozygous and compound heterozygous variants with p.Arg49His in association with p.Phe531Cys a complex heterozygous mutation in family A, and careful follow-up of this family is necessary to elucidate whether the combinations of the variants are related to disease severity. Because of the limited cases of ARVC, further clarification of the proportion of patients with multiple mutations and their respective genotype–phenotype correlations remain to be explored.

## Supplementary information

Supplementary Information.

## Data Availability

The relevant data from this Data Report are hosted at the Human Genome Variation Database at 10.6084/m9.figshare.hgv.2597 10.6084/m9.figshare.hgv.2600.
