# Supplementary material for: Two pedigrees with arrhythmogenic right ventricular cardiomyopathy linked with R49H and F531C mutation in DSG2
Source: Hum Genome Var. 2019 Aug 21;6:38. doi: 10.1038/s41439-019-0069-3 (PMC6804664; doi:10.1038/s41439-019-0069-3)
Supplement: Supplementary file 1 — Supplementary Information. [file 41439_2019_69_MOESM1_ESM.pdf]

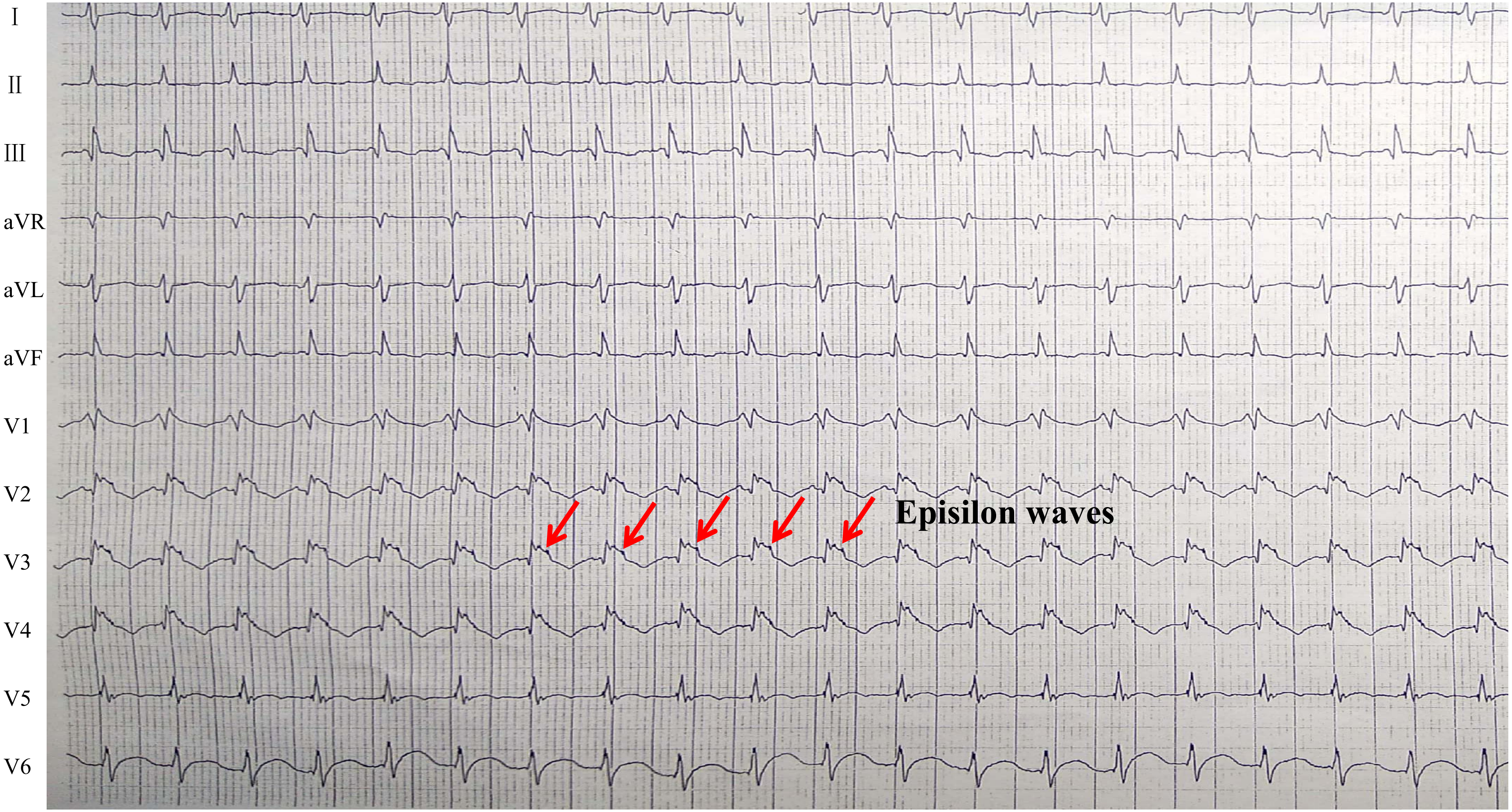

Supplemental Fig. S1 A nodal tachycardia and Episilon waves (arrows noted) were recorded. Paper speed 25 mm/s,calibration 10mm/mv.

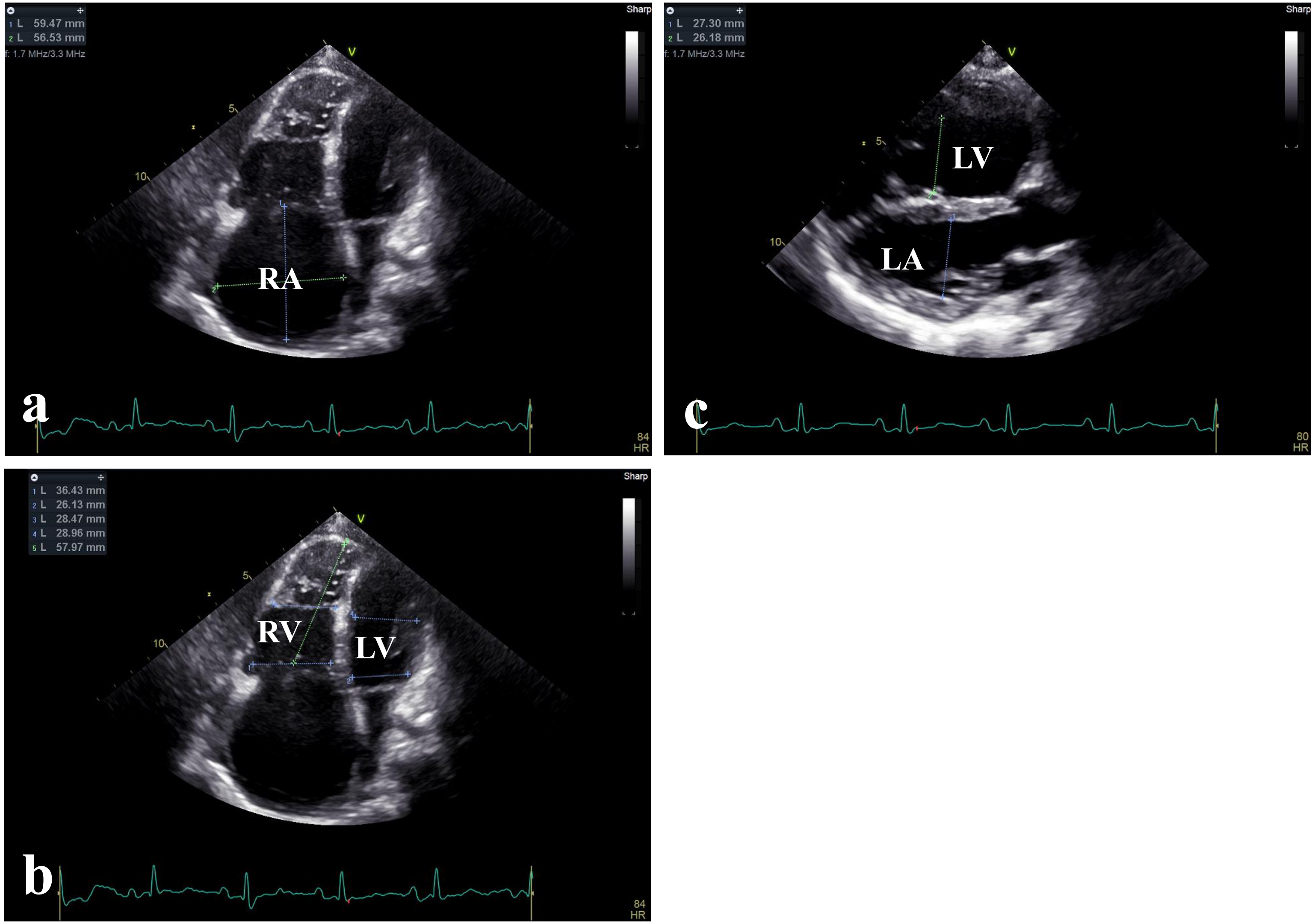

Supplemental Fig. S2 Echocardiograms of the proband (subject III:2), showing dilated RA (c, 59*57mm) and RV (b, basal part, 26mm, partes intermedia, 36mm), normal LV (b, basal part and partes intermedia, 28mm), parasternal long axis view of normal LV (c, 26mm) and LA (c,26mm). RA=right atrial, RV = right ventricle, LV = left ventricle.

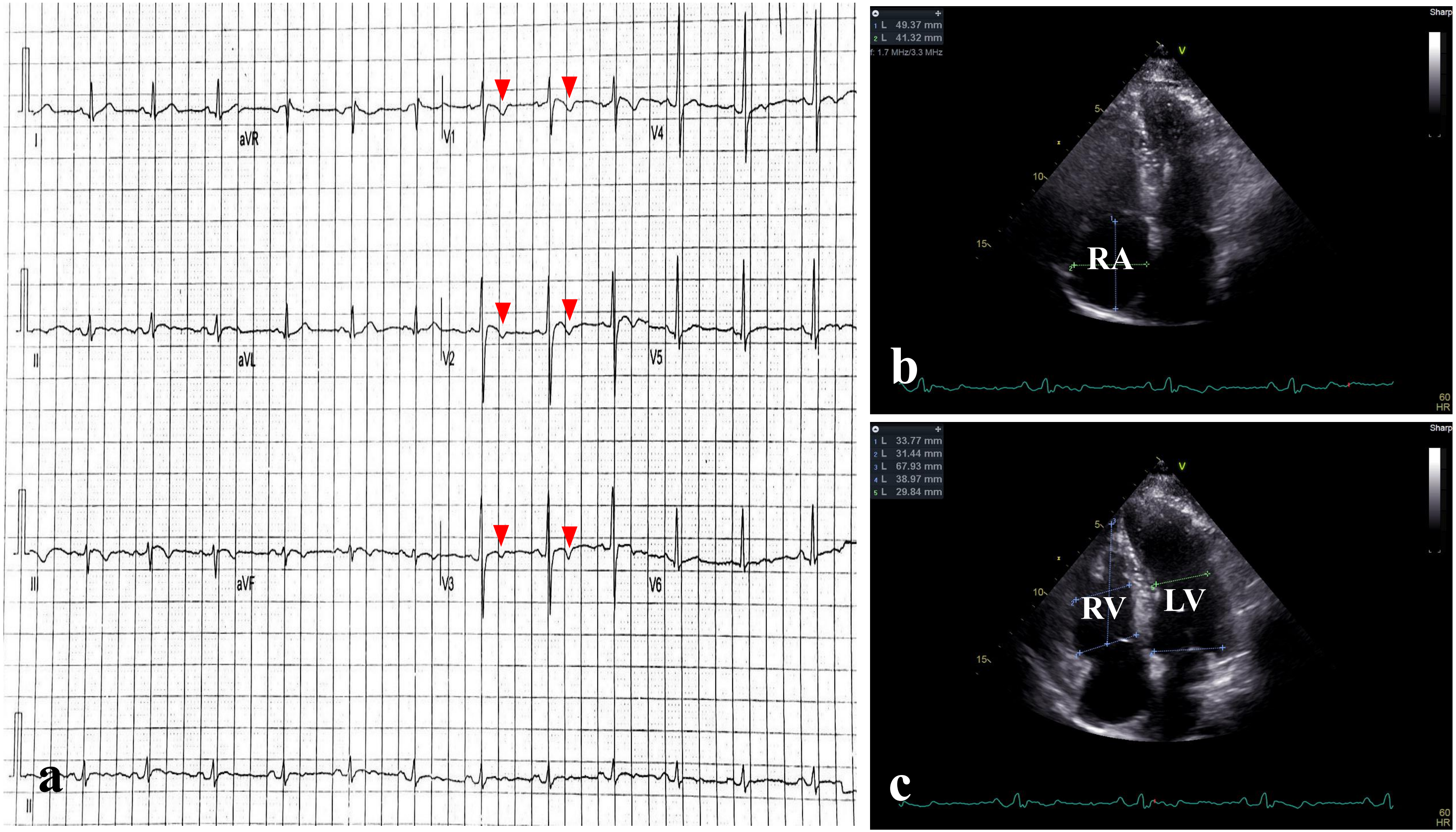

Supplemental Fig. S3 Twelve-lead resting electrocardiogram of the proband's father (subject Ⅱ:3), it shows sinus rhythm at 77 bpm. T-waves inversion from V1 to V3(triangles noted) (a). Paper speed 25 mm/s, calibration 10mm/mv. Apical four chamber view of echocardiograms showing mild dilated RV (c, basal part, 31mm, partes intermedia, 33mm), normal RA (b,41*49mm) and normal LV (c, basal part, 38mm, partes intermedia, 29mm), RA=right atrial, RV = right ventricle, LV = left ventricle.

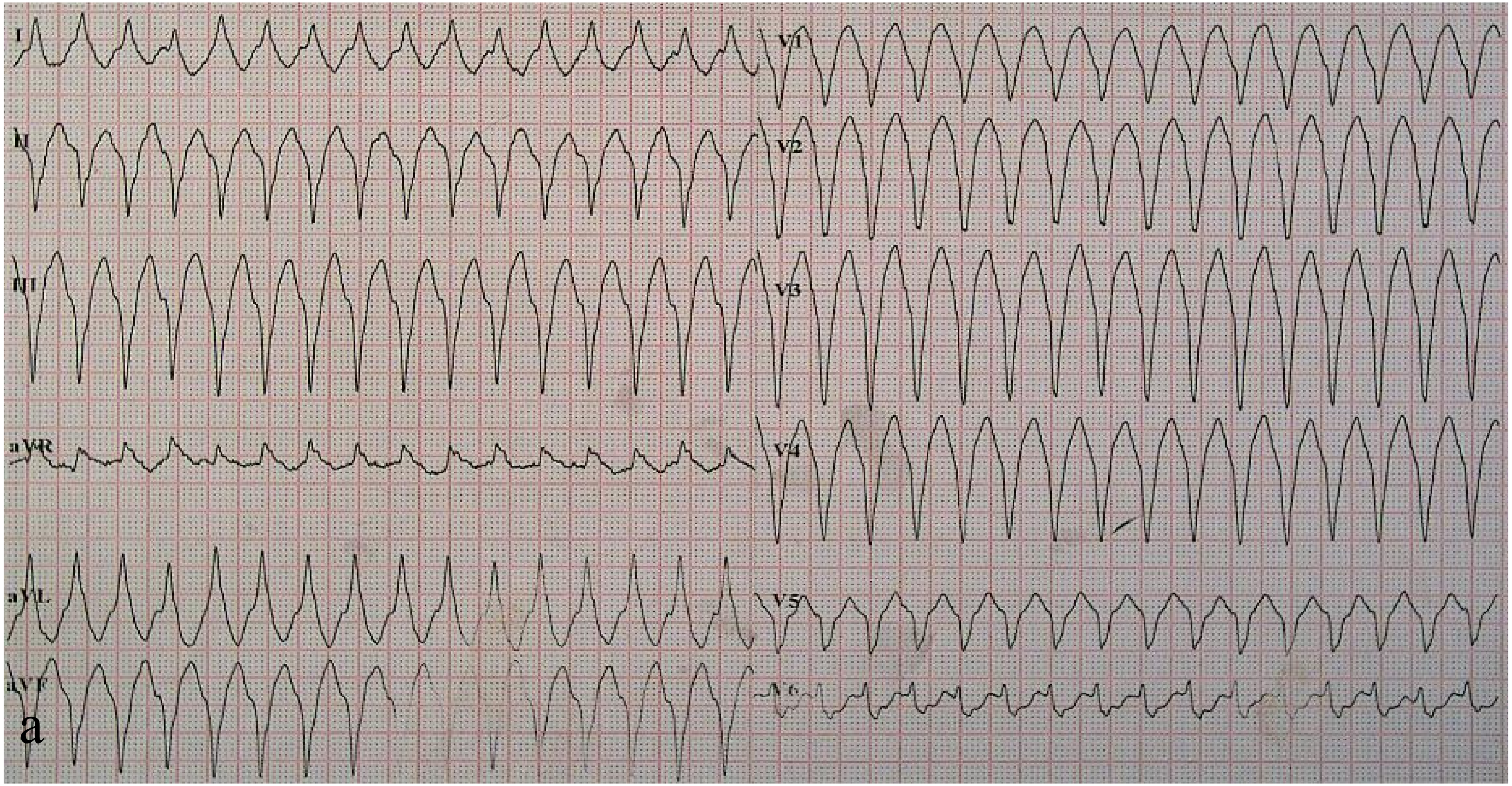

Supplemental Fig. S4 Twelve-lead electrocardiogram of the proband (subject Ⅲ:5, in family B), it shows the sustained ventricular tachycardia Paper speed 25 mm/s, calibration 10mm/mv.

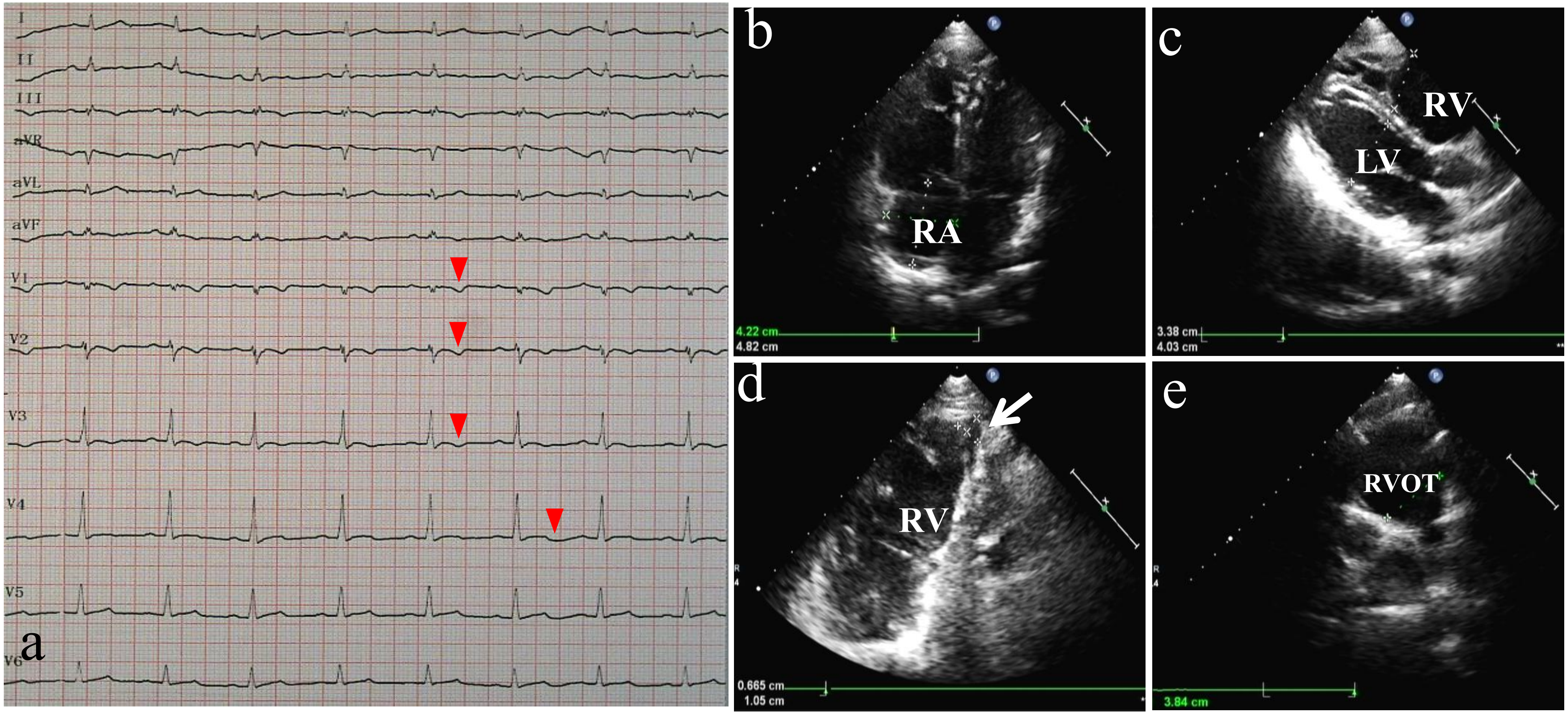

Supplemental Fig. S5 Twelve-lead showed T-wave inversion in chest leads from V1 to V4 (triangles noted)(a). Echocardiograms showing enlarged RA (b,48*42mm), RV(d, 34mm) and normal LV (c, 40mm). Right ventricular apical aneurysm (d, 11*7mm, arrow noted) and broaden right ventricular outflow tract (e, RVOT, 38mm) was shown. RA=right atrial, RV = right ventricle,LV = left ventricle.
